# Perioperative Anesthesiological Management of Patients with Pulmonary Hypertension

**DOI:** 10.1155/2012/356982

**Published:** 2012-10-12

**Authors:** Jochen Gille, Hans-Jürgen Seyfarth, Stefan Gerlach, Michael Malcharek, Elke Czeslick, Armin Sablotzki

**Affiliations:** ^1^Klinikum St. Georg gGmbH, Klinik für Anästhesiologie, Intensiv und Schmerztherapie, Delitzscher Straße 141, 04129 Leipzig, Germany; ^2^Medical Clinic and Polyclinic I, Department of Pneumology, Universitätsklinikum Leipzig AöR, 04103 Leipzig, Germany; ^3^Clinic for Anesthesiology and Critical Care Medicine, Martin Luther University of Halle-Wittenberg, 06120 Halle, Germany

## Abstract

Pulmonary hypertension is a major reason for elevated perioperative morbidity and mortality, even in noncardiac surgical procedures. Patients should be thoroughly prepared for the intervention and allowed plenty of time for consideration. All specialty units involved in treatment should play a role in these preparations. After selecting each of the suitable individual anesthetic and surgical procedures, intraoperative management should focus on avoiding all circumstances that could contribute to exacerbating pulmonary hypertension (hypoxemia, hypercapnia, acidosis, hypothermia, hypervolemia, and insufficient *anesthesia and analgesia*). Due to possible induction of hypotonic blood circulation, intravenous vasodilators (milrinone, dobutamine, prostacyclin, Na-nitroprusside, and nitroglycerine) should be administered with the greatest care. A method of treating elevations in pulmonary pressure with selective pulmonary vasodilation by inhalation should be available intraoperatively (iloprost, nitrogen monoxide, prostacyclin, and milrinone) in addition to invasive hemodynamic monitoring. During the postoperative phase, patients must be monitored continuously and receive sufficient analgesic therapy over an adequate period of time. All in all, perioperative management of patients with pulmonary hypertension presents an interdisciplinary challenge that requires the adequate involvement of anesthetists, surgeons, pulmonologists, and cardiologists alike.

## 1. Background

Pulmonary hypertension represents an important risk factor for increased perioperative morbidity and mortality. Stress, pain, ventilation, and surgery-related inflammation can further increase pressure and resistance within the pulmonary arteries and cause right-sided heart failure. Ramakrishna et al. have described a number of independent factors leading to an increased perioperative risk for patients with pulmonary hypertension. Conditions that caused one or more perioperative complications in 42% of all patients were heart failure of NYHA class II or higher, a history of pulmonary embolism, high-risk surgery (e.g., thoracic or major abdominal surgery), and an anesthesia duration of more than 3 hours [1]. The literature reports a perioperative mortality of 7–24%—depending on the primary disease and the type of surgical intervention—with the highest risk for pregnant women and patients undergoing emergency interventions [1–5].

In a recently published study, Kaw et al. examined the clinical progression of 96 patients with pulmonary hypertension who underwent a noncardiac surgical procedure. The PH patients had a significantly increased risk for hemodynamic instability, heart failure, postoperative sepsis, and respiratory failure. In addition, they required significantly prolonged postoperative ventilation and a longer intensive care stay and had to be readmitted for inpatient treatment much more frequently within the first 30 days following surgery [6].

Perioperative management of patients with major pulmonary hypertension also presents a great challenge to physicians. In the opinion of the authors, it is crucial to understand the pathophysiological mechanisms underlying this disease, not only for the long-term treatment of patients with pulmonary hypertension, but also for the development of treatment concepts in the perioperative environment. The anesthetist will have a central role to play in achieving this objective. Consequently, this paper will begin by what is currently known about the pathophysiology of pulmonary hypertension and right-sided heart failure, before outlining concepts for perioperative *anesthesiological* management *focusing on noncardiac surgery*.

## 2. Definition and Pathophysiology of Pulmonary Hypertension

Pulmonary hypertension comprises a number of diseases, all of which have the common symptom of increased pressure in the pulmonary arteries. These diseases are characterized by a progressive course and a poor prognosis for the patient (Dana Point Classification; see Table 1) [7]. In order to diagnose pulmonary hypertension (PH), it is necessary to measure the mean pulmonary artery pressure (PAPm) during right-heart catheterization. PH is defined as PAPm > 24 mmHg at rest. Values of 20 ≤ PAPm ≤ 24 mmHg are referred to as borderline PH [8, 9]. Mean pulmonary artery pressure in healthy persons is 14 ± 3 mmHg [10].

There are multiple reasons for elevated pressure in the pulmonary circulation. A basic distinction is made between precapillary and postcapillary PH. Postcapillary PH is caused by left-heart disease, whereas the various forms of precapillary pulmonary hypertension are differentiated on the basis of their origin (Tables 1 and 2).

In many cases, the pulmonary pressure elevation is a result of left-heart disease (→ group 2, Tables 1 and 2), which triggers “back pressure” effects in the veins and consequently an elevation in pulmonary artery pressure. This causes reactive changes of the vascular bed supplying the lung, accompanied by vasoconstriction, remodeling of the pulmonary vessels [11], and consequently an increase in the transpulmonary pressure gradient (TCG = PAPm-PCWP). For chronic pulmonary diseases (→ group 3, Tables 1 and 2), however, a large number of pathomechanisms may increase the pressure in the pulmonary vessels, either individually or in combination. The main inducers are hypoxic vasoconstriction and a disequilibrium between vasodilating (NO, prostaglandins) and vasoconstricting (thromboxane, endothelin) mediators. In the event of pulmonary fibrosis and emphysema, there is an additional loss of the capillary bed. Chronic thromboembolic pulmonary hypertension (CTEPH; → group 4, Tables 1 and 2) may also develop and lead to partial or complete occlusion of the pulmonary arteries through the formation of mural thrombi. It is interesting to note that, in the case of CTEPH, the remodeling processes described previously also occur in vessel regions not affected by thrombi. In addition, increased pulmonary pressure also occurs in many other diseases (→ group 5, Tables 1 and 2) that are caused by mechanisms [that are as yet unspecified and/or multifactorial [8].]

Pulmonary arterial hypertension (PAH; → group 1, Tables 1 and 2) is a primary disease of the small pulmonary arteries and arterioles, which, in terms of vascular remodeling, includes all layers of the affected vessels, both functionally and structurally [11]. The reasons for this disease are multifactorial and include genetic, endothelial, inflammatory, immunological, and coagulatory factors [8]. In idiopathic pulmonary arterial hypertension (IPAH), the modifications to the pulmonary arteries described previously occur even in the absence of the usual trigger mechanisms.

**Figure 1:**
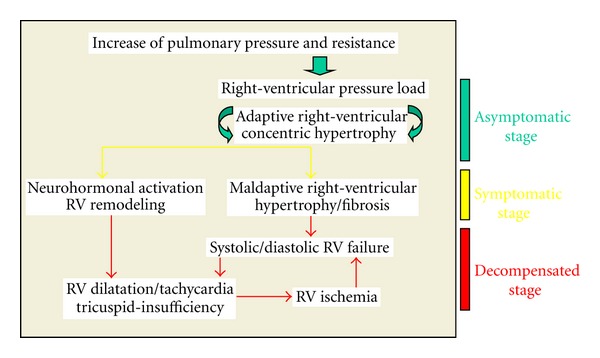
Development of right-ventricular failure in patients with pulmonary hypertension.

The increased pulmonary pressure and resistance, in combination with the chronically elevated afterload, result in hypertrophy and dilation of the right ventricle. A phase of stable physiological adaptation (remodeling of the right ventricle) is followed by maladaptive hypertrophy and progressive right-sided heart failure with decreased cardiac output, even at rest, and the typical clinical symptoms (Figure 1, Table 3). In this case, the degree of right-ventricular dysfunction is critical in determining the prognosis of the disease.

## 3. Prevalence of Pulmonary Hypertension

There is no reliable data on the prevalence of patients with PH, for example, in an average anesthesiological patient population. An impression can be gained by considering the prevalence of individual entities of PH. In a major study in France, a prevalence of 15/1 million inhabitants (5–25/1 million including regional variations) has been observed for pulmonary arterial hypertension (PAH; → group 1, Tables 1 and 2), whereas the figure for idiopathic pulmonary arterial hypertension (IPAH) is about 5/1 million inhabitants [12]. Pengo et al. give a prevalence of 1–4% for pulmonary hypertension developing in patients who are survivors of pulmonary embolism (CTEPH; → group 4, Tables 1 and 2) [13].

The occurrence of PH in restrictive and obstructive lung diseases varies, depending on the severity of these diseases. In a collective of 215 patients with severe chronic obstructive pulmonary disease or pulmonary emphysema who underwent right-heart catheterization before a planned lung transplantation or lung volume reduction, 50.2% of patients (*n* = 108) had a PAPm > 25 mmHg, but only 3.7% (*n* = 8) had a PAPm > 45 mmHg [14]. A cluster analysis of these patients showed that a small subpopulation had an insufficiently high PAPm (38.8 mmHg), even though their lung function was only moderately limited (FEV1: 48.5%). Under certain conditions (PAPm > 35; PAPm > 25 and cardiac index < 2.0 1/min/m²; pulmonary vascular resistance >480 dyn∗s∗cm^−5^, at least 2 out of 3 criteria), it must be assumed that IPAH also exists independently of PH in these cases [15].

*This underlines* that the occurrence of an IPAH severely affecting hemodynamics is rare and, in most cases, has already been detected. On the other hand, our hospitals are confronted much more frequently with the disease pattern of all forms of PH on a daily basis.

## 4. Treatment of Pulmonary Hypertension

The therapeutic approach is guided by the diagnosis of the PH type. In recent years, various new pulmonary vasodilators have been successively tested for their effects in clinical trials and launched on the market. However, these drugs are only approved for the therapy of PAH (→ group 1, Tables 1 and 2). They exert their effects using different signaling pathways, that is, the *endothelin-signaling pathway* (endothelin receptor antagonists: bosenthan, ambrisentan), the prostacyclin-signaling pathway (prostacyclin analogues: iloprost, epoprostenol, and treprostinil), and the NO*-signaling pathway* (phosphodiesterase-5 inhibitors: sildenafil, tadalafil).  Table 4 gives an overview of all approved substances [8].

The most potent vasoconstrictor in the human organism is endothelin-1 [16]. It exerts an influence on the pulmonary arteries by means of two receptors—the endothelin-A receptor and the endothelin-B receptor. There is a local difference in the distribution of the two receptors, which is modified under the influence of pulmonary hypertension. The dual endothelin receptor antagonist bosentan is approved for the treatment of PAH patients in WHO functional classes II and III. Its effectiveness was confirmed for patients with idiopathic or familial PAH and for PAH associated with connective tissue disease or congenital heart defects. In the study leading to the substance's approval, the *capacity *of the patients (*n* = 144, bosentan of either 125 mg or 250 mg/d; *n* = 69 placebo) improved after 16 weeks of treatment, as measured by an average improvement of 36 m in the six-minute walk distance (6MWD) [17]. An elevation in transaminases was observed in 7-8% of patients treated with bosentan, which eventually led to a therapy interruption in approx. Three percent of all patients. For this reason, it is essential to monitor transaminases during therapy (every 4 weeks) [18]. 

Similar regulations apply to the approval of ambrisentan, a selective endothelin-A receptor antagonist. A study examining ambrisentan administered in a dose of 5 or 10 mg compared to a placebo in patients with idiopathic PAH or PAH with systemic sclerosis observed an improvement of 31 m (5 mg) or 51 m (10 mg) in the 6 MWD [19]. Although this drug is not associated with hepatotoxicity, the formation of peripheral edema is often observed during ambrisentan therapy. 

Two phosphodiesterase-5 inhibitors, sildenafil and tadalafil, are available for the treatment of pulmonary arterial hypertension. They elevate cGMP by blocking its decomposition, thereby inhibiting calcium entry and consequently enabling pulmonary vasodilation. The formulation of its indication is similar to that of the endothelin antagonists. Meanwhile, data is now available in relation to monotherapy with sildenafil over 3 years. A survival rate of 79% was observed among patients in this study (*n* = 259). After 3 years of therapy, 60% of patients showed the same or better results in relation to walk distance in the six-minute walk test and their WHO functional class [20]. It is noteworthy that the majority of patients received a dose (3 × 80 mg) above the approved dose (3 × 20 mg). In a double-blind placebo-controlled prospective study with the approved dose (1 × 40 mg) over 16 weeks, tadalafil led to an improvement of 33 m in 6MWD. This dose also had a significant positive influence on other endpoints, for example, on the time until clinical aggravation [21]. New drugs elevating the cGMP level through stimulation of guanylyl cyclase are currently undergoing clinical trial, and the initial results look promising. 

In contrast, the prostanoids reduce calcium entry into smooth muscle cells by elevating the cAMP level. They also have an influence on endothelial cells, thrombocytes, leukocytes, and fibroblasts. Two drugs from this group are approved in Germany, that is, inhalative iloprost and long-term subcutaneous treprostinil. Their indication is limited to idiopathic pulmonary arterial hypertension for patients in WHO functional class III. It has long been known that inhaled prostanoids reach the affected organ directly (selective pulmonary vasodilation), which minimizes systemic side effects. In 2002, Olschewski and coworkers investigated daily inhalation of iloprost in a large randomized placebo-controlled multicenter study and showed a significant increase in the distance walked in six minutes, a significant improvement of hemodynamic values, an improvement in the NYHA-class, dyspnea, and quality of life [22]. Inhalative application also offers the option of treating ventilated patients using a dedicated nebulizing system. 

Long-term subcutaneous administration of treprostinil is realized using a subcutaneous catheter with a supply line and a pump. However, three quarters of patients experience pain in the injection site when receiving this treatment. The only drug that was recently approved for the treatment of IPAH in Germany is epoprostenol. In spite of the difficulties caused by its method of administration and the short half-life of epoprostenol, it is still the most frequently used prostanoid worldwide for intravenous application. A major study of 162 IPAH patients being treated with epoprostenol showed 1-, 2-, and 3-year survival rates of 88%, 77%, and 63% [23].

No drugs for pulmonary vasodilation have been approved for the treatment of other forms of PH outside of group 1 (Tables 1 and 2). For thromboembolic pulmonary hypertension in particular, operative thromboendarterectomy is the treatment of choice and should always be considered. Prospective randomized studies for this indication have been unable to show any clear benefit of a specific pulmonary vasodilator treatment [24]. Nonetheless, there are indications that some of these patients could benefit from drugs originally approved for PAH [25].

Basic therapeutic measures for PH, especially in the case of stress or partial respiratory insufficiency, are oxygen supplementation and, if signs of right-sided heart failure are detected, diuretic therapy. It is also advisable to administer anticoagulants to patients with idiopathic, familial, or thromboembolic pulmonary hypertension [8].

## 5. Preoperative Preparation and Diagnostics: An Interdisciplinary Task

Given that pulmonary hypertension affects several organ systems simultaneously (lung, heart, and vascular system), preparations for the surgical procedure should be considered as the joint task of anesthesia, surgery, pulmonology, and cardiology [1, 26]. The purpose of these preparations should be, on one hand, to evaluate the functional state of the heart and lung organ systems as good as possible so that the probability of complications, including right-sided heart failure, can be properly estimated. On the other hand, experts should strive to optimize the patient's initial condition as far as possible by adjusting the current specific medication and treatment of comorbidities, which minimizes the individual risk of complications [27]. In many cases, patients with pulmonary hypertension receive ongoing anticoagulant therapy, which necessitates the adaptation and preparation of anticoagulant medication prior to inpatient hospitalization. This includes establishing, prior to the surgical procedure, if postoperative recovery at home is an option and if the prerequisites for this option can be fulfilled (family environment, primary care physician, social services, outpatient services, and insurance providers) [27].

### 5.1. Clinical Examination

**Figure 2:**
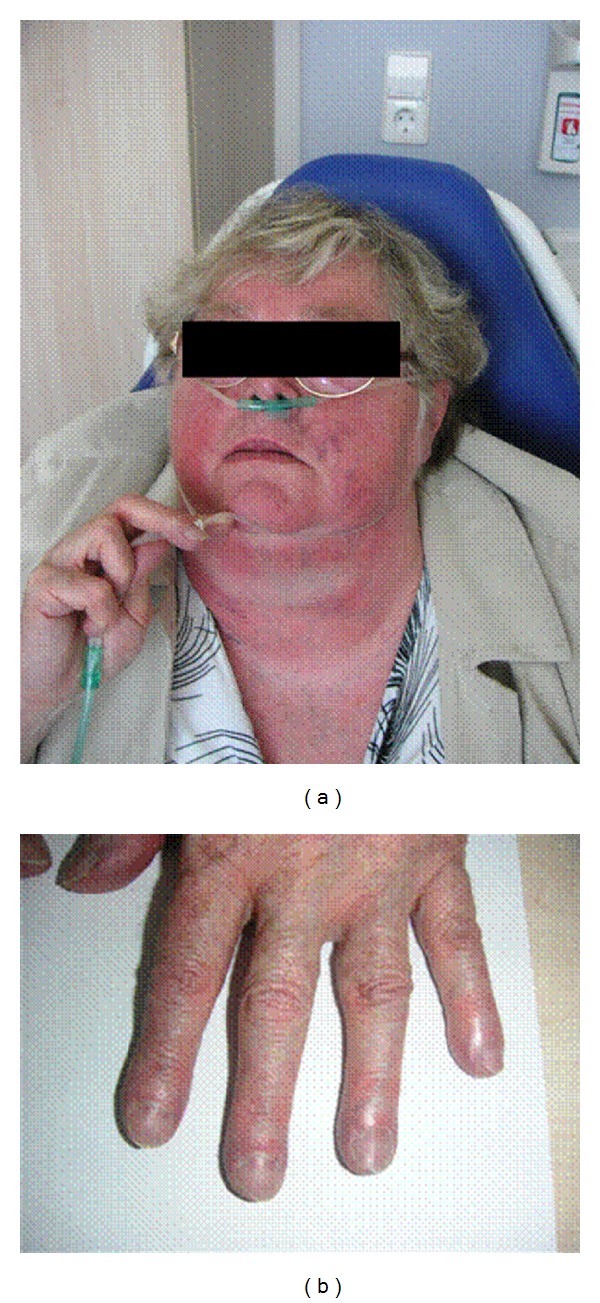
Clinical findings in a patient with chronic right-heart insufficiency and severe pulmonary hypertension.

The clinical symptoms of pulmonary hypertension are largely unspecific and easily overlooked or misinterpreted in the early disease stages (Table 3). The most common but, unfortunately, very unspecific symptom is stress-induced dyspnea. In addition to obtaining a detailed medical history, the clinical investigation should focus on symptoms for right-sided heart failure. In late-stage diseases in particular, obstruction of the jugular veins, peripheral edema, hepatomegaly, hepatojugular reflux, and ascites are probable (Figure 2).

The stages of pulmonary hypertension are divided into four functional classes according to the criteria of NYHA/WHO (Table 5) [28].

### 5.2. Thoracic X-Ray

Characteristic findings for pulmonary hypertension include right-ventricular hypertrophy, dilation of the central pulmonary arteries, and vascular rarefaction in the peripheral pulmonary parenchyma [8].

### 5.3. ECG

**Figure 3:**
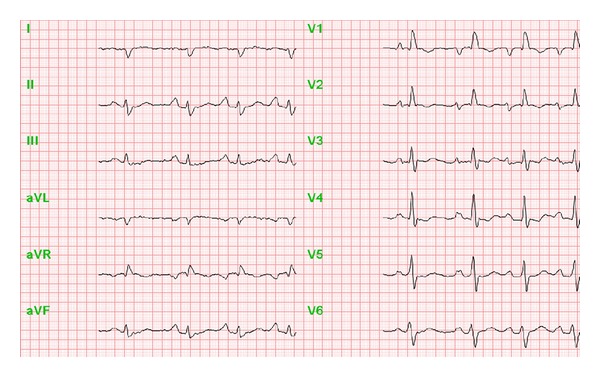
ECG in a patient with severe pulmonary hypertension.

In the ECG, characteristic changes are also more pronounced if major right-ventricular load already prevails (Table 6, Figure 3). Typical symptoms for pulmonary hypertension are right bundle branch blocks and “snow-shovel-” shaped ST segment depressions in the precordial leads [8].

### 5.4. Pulmonary Function Examination

It is recommended, in particular for patients with chronic lung disease as a cause of pulmonary hypertension, that an examination of pulmonary function and an arterial blood gas analysis be conducted prior to the surgical intervention [27].

### 5.5. Spiroergometry

Important information for estimating the severity and progression of the disease can be obtained using spiroergometry [8]. The most important parameters are the maximum oxygen uptake (peak VO_2_), the ventilatory equivalent ratio for CO_2_ (VE/VCO_2_), and the partial pressure of end-tidal carbon dioxide (PETCO_2_).

### 5.6. Echocardiography

Echocardiography is currently the noninvasive method with the highest sensitivity and specificity for diagnosing PH [29]. Furthermore, echocardiography is suitable for monitoring the disease course and establishing a prognosis. Independent prognostic factors are an enlarged right atrium (RA surface > 27 mm²), the presence of pericardial effusion, and impaired global pumping capacity of the right ventricle [29]. Through echocardiography, it is possible to diagnose a left-ventricular dysfunction, valvular insufficiencies, or shunts as reasons for PH. However, note that echocardiography cannot replace right-heart catheterization for making a definitive diagnosis of PH [8].

### 5.7. Right-Heart Catheterization

Right-heart catheterization is a direct means of measuring the hemodynamic parameters in pulmonary circulation and obtaining important information about the severity of pulmonary hypertension and right-ventricular functionality [8]. For patients with late-stage pulmonary hypertension, current hemodynamic data (not older than 3-4 months) should be available at the time of the surgical intervention. If necessary, a repeat examination should be carried out (at the very least in the case of an aggravation of the clinical condition). The findings of right heart catheterization provide important leads for evaluating the range of hemodynamic parameters in the perioperative course and for determining the point at which therapeutic measures should be initiated [2]. The parameters to be determined are listed in Table 7.

For intra- and postoperative treatment options, it is important to know if responsiveness to inhaled vasodilators such as *nitric oxide* (NO) or iloprost is assured, as both substances can be administered for the selective therapy of an acute increase in pulmonary artery pressure (see also intraoperative management) [30].

### 5.8. Optimization of Primary Disease Therapy

Before surgical intervention, medication should be critically examined from a pulmonological and cardiological perspective and under consideration of recent hemodynamic findings, with a view to possible optimization. This applies, in particular, to the specific therapy of pulmonary hypertension. At the time of surgery, the patient should ideally have been in a stable condition for an extended period of time [27, 30].

If the pulmonary hypertension was discovered immediately prior to urgent surgery that could not be postponed, Fox et al. recommended starting treatment with sildenafil (50–100 mg/day) and L-arginine (15 g/day) as early as possible [31]. We do not support this concept and would like to modify this recommendation by proposing that a preoperative differentiation should be made between the pre- and postcapillary genesis of PH, which, in most cases, can quickly be made on the basis of an echocardiography *and/or right-heart catheter*.

### 5.9. Risk Disclosure

Given that patients with pulmonary hypertension suffer significantly increased perioperative morbidity and mortality, a perioperative assessment of risks and possible benefits of surgical intervention plays a critical role. For this reason, patients should, whenever possible, be thoroughly informed about possible risks long before surgery in order to give them sufficient time for consideration. Close family members should also be involved if possible.

Risk disclosure involves explaining necessary preoperative tests, presenting intra- and postoperative monitoring methods (pulmonary artery catheterization, TEE, etc.), a thorough description of surgical and anesthetic procedures and their respective risks, recovery (intensive and/or intermediate care unit stay), as well as possibilities of postoperative analgesic therapy. Depending on the initial condition of each patient, they may also need to be explicitly informed about the possibility of severe complications that can lead to extended hospitalization or even death.

### 5.10. Intraoperative Monitoring

For patients with pulmonary hypertension, intraoperative monitoring should be adapted to the severity of the disease and the invasiveness of the surgical procedure, although, to date, there is no evidence to suggest that any specific type of monitoring has an influence on patient morbidity and mortality. However, the authors believe that early recording of deviations from the initial condition (in particular in relation to right-heart function) can make a decisive contribution to recognizing and avoiding severe complications from the outset.

Whereas basic monitoring can be considered sufficient for minor and medium procedures in functional state II, all major interventions and those in functional state III should be carried out under an extended monitoring (Table 8). Invasive blood pressure measurement is a basic procedure for patients with pulmonary hypertension, in addition to standard anesthesiological monitoring (continuous ECG, SaO_2_ and end-expiratory CO_2_). Intermittent blood gas analysis should also be carried out using an arterial catheter. Fox et al. recommend measuring the central venous pressure (CVP) for intraoperative volume control [31]. However, other forms of examination—although not yet specifically evaluated for patients with pulmonary hypertension—have shown that stroke volume variability (SVV) is a much more appropriate method of evaluating volume responsiveness, provided that the prerequisites for its use are fulfilled (sinus rhythm and ventilation) [32].

For all patients in the late stages of the disease and with existing right-sided heart failure, pulmonary artery catheterization and transesophageal echocardiography (TEE) are the methods for an adequate intraoperative monitoring of right-heart function and visualization of the required therapy effects if necessary [30]. TEE offers the opportunity for several important assessments: left-ventricular end-diastolic area in the transgastric short-axis view (normal values: >5.5 cm²/m² and <11.9 cm²/m²), identification of myocardial ischemia by segmental evaluation of left ventricular wall thickening, color Doppler monitoring of all valves, color Doppler monitoring of pulmonary artery systolic pressure and tricuspidal regurgitation, and continuous Doppler measurements for calculation of cardiac output [33]. The usefulness of intraoperative TEE even in noncardiac surgery is supported by the studies of the European Perioperative Transesophageal Research Group, who showed that the use of TEE caused a significant change in overall therapeutic management in 30% of patients [33, 34]. In patients with pulmonary hypertension and an impaired right-heart function, timing and amount of fluid therapy are crucial points for the intraoperative management. Intraoperative TEE may be an important tool for the optimization of intraoperative fluid therapy; Hofer and coworkers investigated 99 high-risk noncardiac surgical patients and found that TEE caused changes in administration of fluids in 47% [35]. It is important to notice that TEE may be advantageous compared to the monitoring by pulmonary artery catheter (PAC); Poelaert et al. assessed the impact of TEE on therapeutic management in relation to PAC in 103 critically ill patients and found that TEE modified therapeutic changes in 30% of these patients, despite the presence of a PAC [36]. But there are several factors limiting the routine intraoperative use of TEE. The handling and interpretation of findings is dependent from the training and personal experience of the investigator, and the method is not tolerated in awake patients undergoing regional anesthesia [33].

The intraoperative use of pulmonary artery catheters is subject to controversial discussions in the current literature. Whereas Rinne and Zwissler rarely found it necessary [37], Fox et al. [31], Subramaniam and yared [26], and Krug et al. [38] believe that continuous monitoring of pulmonary artery pressure is essential to the regulation of intraoperative therapy. In addition to continuous measurement of mean pulmonary artery pressure (mPAP), systemic and pulmonary resistance and the cardiac index may act as useful indicators for controlling volume substitution and the administration of vasodilating or inotropic medication [31]. However, all authors point out that the insertion of a pulmonary artery catheter is associated with certain risks, which must be considered when applying this monitoring method.

As the central venous saturation (ScvO_2_) is a surrogate of the mixed venous saturation (SvO_2_), it reliably can be used as a marker of global tissue perfusion and may represent an alternative way of intraoperative monitoring. Studies from cardiac surgery have shown that there is a predictive power of intermittently measured ScvO_2_ for adverse events [39]. But anesthetists should know about the factors influencing ScvO_2_ values intraoperatively and therefore limit the validity for intraoperative decision making. A decrease in ScvO_2_ may be caused by (a) a decrease in SaO_2_, (b) a decrease in cardiac output, (c) a decrease in hemoglobin level, and (d) an increase of oxygen consumption [40].

### 5.11. Selection of the Anesthetic Technique

At this point, it is important to emphasize that the selection of the anesthesia team is just as crucial as the selection of the anesthetic technique to be used. It is essential to have not only excellent anesthesiological expertise on hand, but also specific knowledge of the pathophysiology of pulmonary hypertension and right-sided heart failure, the interpretation of hemodynamic data, and the corresponding concepts of complex medical treatment. Ideally, it should be possible to carry out transesophageal echocardiography and interpret its results. In particular, patients with late-stage pulmonary hypertension should be treated in medical centers that fulfill all conditions for qualified treatment in terms of their structure and personnel (Table 9).

All standard anesthetic techniques can, in principle, be applied to patients with pulmonary hypertension [31, 36, 38]. Regional anesthetic techniques offer the advantage of not impairing spontaneous breathing and of avoiding elevated pulmonary pressure, which is induced by mechanical ventilation [41, 42]. In addition, techniques of continuous regional anesthesia can be used for postoperative analgesic therapy. In general, continuous techniques should be preferred to bolus administration—especially for procedures in the vicinity of the spinal cord—in order to avoid uncontrolled drops in blood pressure, which can endanger myocardial perfusion. During induction of epidural analgesia, administration and dosing of local anesthetics should be performed carefully and fractionated in order to avoid significant decrease in systemic vascular resistance, reduction of coronary perfusion, and right-heart failure. Due to this risk, spinal anesthesia with bolus technique should be avoided and also replaced by a catheter technique to avoid any significant effects on hemodynamics [27, 39]. Plexus catheters or nerve catheters (sciatic or femoral nerve) are recommended for surgical procedures involving the extremities in particular, as they do not affect hemodynamics, have a low failure rate, and ensure treatment of postoperative pain. Techniques in the vicinity of the spinal cord can be applied both in hernia surgery and in urological or gynecological interventions. In abdominal and thoracic surgery in particular, it is advisable to combine general and thoracic epidural anesthesia in order to reduce intraoperative consumption of anesthetic agents and to avoid high doses of systemic opioid analgesics in the postoperative phase. Thoracic epidural anesthesia does not have any significant influence on oxygenation and pulmonary vascular tension [35, 41]; it should be noted, however, that a high ratio (TH1-TH4) can cause sympathetic blockade and therefore a decrease in myocardial inotropy and chronotropy [36, 43–45]. In the context of obstetrics, the successful application of lumber epidural anesthesia has repeatedly been described [46], even though more recent literature stresses the higher morbidity of pregnant women with pulmonary hypertension (up to 36%) and the significance of a multiprofessional approach in perioperative treatment [47].

It should be considered that surgical interventions under spontaneous breathing and regional anesthesia may prove difficulty if intraoperative positioning with the slightly elevated upper body is not possible. Particularly in the later stages of pulmonary hypertension or in the case of severe primary diseases affecting the lung, patients cannot be subjected to remain in a flat position over a long period of time. In these cases, regional anesthesia must nevertheless be administered. It is recommended that this technique be combined with general anesthesia to ensure adequate oxygenation. 

Nearly all patients with pulmonary hypertension receive continuous anticoagulant therapy, and this fact must be given special consideration when planning the intervention and the selected regional anesthetic procedures. Recommendations in relation to this issue are provided in the current guidelines on “regional anesthetic procedures in the vicinity of the spinal cord and thrombosis prevention/antithrombotic medication” [48, 49].

**Figure 4:**
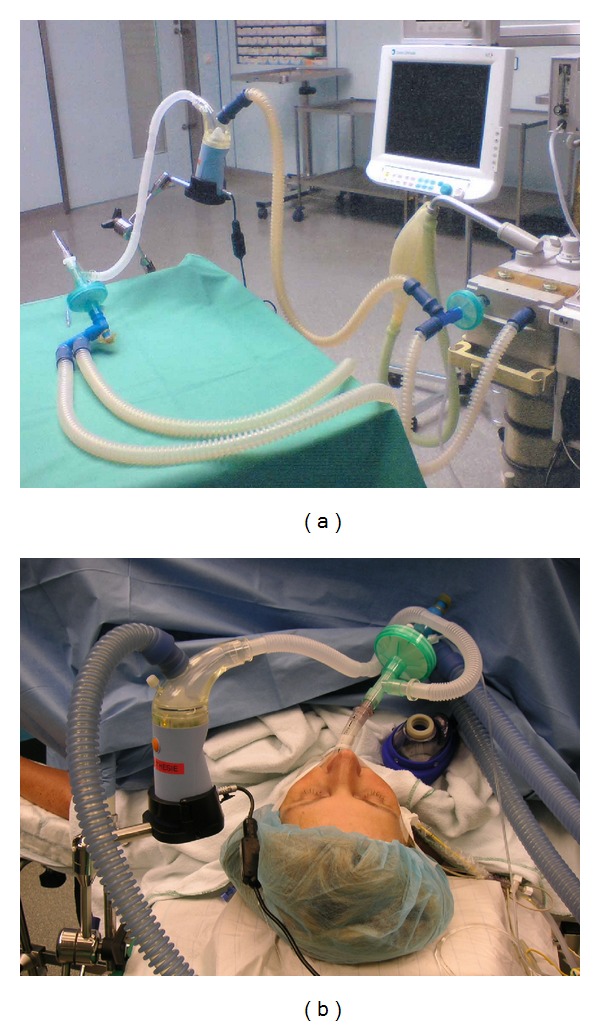
Assembly instruction for the integration of an ultrasonic nebulizer (Multisonic) in the ventilatory circuit. Intraoperative selective pulmonary vasodilation with inhaled iloprost.

The main advantages of general anesthesia are safe oxygenation and uncomplicated airway management. When the patient is intubated during anesthesia, inhalants for selective pulmonary vasodilation can easily be administered through the breathing gas (Figures 4(a) and 4(b)). A disadvantage lies in the stage of anesthesia introduction, which is often accompanied by extreme blood pressure variations. The combination of anesthetic-agent-induced systemic vasodilation and mechanical ventilation can lead to a significant drop in mean arterial pressure, which has the potential to *reduce coronary perfusion pressure* and critically affect right-ventricular contractility [31, 41, 50].

For these reasons, a “gentle” introduction phase that avoids arterial hypotension is vital, in particular for patients with pulmonary hypertension. All standard induction agents (recommended doses: thiopental 2–5 mg/kg KG, propofol 1-2 mg/kg KG, and etomidate 0.2–0.4 mg/kg KG) can, in principle, be used in combination with opioids (recommended doses: fentanyl 5–10 *μ*g/kg KG and sufentanil 0.5–1 *μ*g/kg KG), as they have no influence on pulmonary vascular resistance and oxygenation [50–52]. Histamine-releasing relaxants (atracurium, mivacurium) should be avoided for patients with pulmonary hypertension, as they may further increase pulmonary resistance [41, 50].

Nearly all inhalational anesthetics block ATP-dependent potassium channels whose activation induces vascular relaxation. Some time ago, a working group headed by Murray succeeded in demonstrating this in an animal model (dogs) for isoflurane, desflurane, and enflurane, but not for sevoflurane [53, 54]. Some of these experimental results conflict with the different experiences from clinical practice, mainly concerning patients under one-lung ventilation. To summarize, the data suggests that volatile anesthetic agents of concentrations up to 1 MAC can be administered without any negative effects on pulmonary pressure and resistance [27, 30, 37, 41]. In this context, many authors suggest a balanced technique, mixing opioids and low-dose volatile anesthetic agents, which can also be used for maintaining anesthesia [41, 50]. Currently the administration of ketamine and nitrous oxide is not recommended for patients with pulmonary hypertension [31, 37, 50]. Several authors reported an increase of PAPm and/or PVR after injection of ketamine in adults and in children as well as after inhalation of nitrous oxide [55–57]. In contrast to the—mostly older—observations of ketamine effects, Williams and coworkers published in 2010 a retrospective analysis of 68 children who received ketamine for 149 minor and major surgical or diagnostic procedures. The administration of ketamine was not associated with increased complications [58].

### 5.12. Intraoperative Management and Treatment of Intraoperative Pulmonary Artery Pressure Elevation

**Figure 5:**
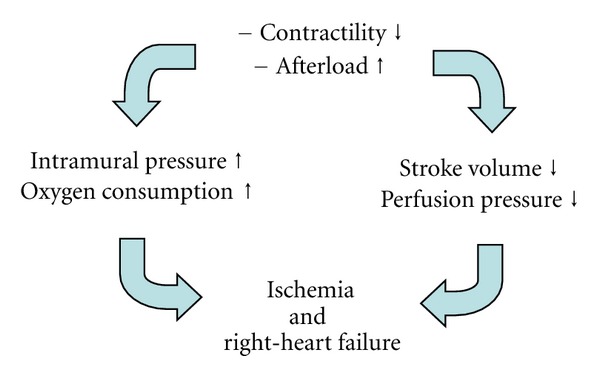
Mechanisms of acute right-heart failure.

The particularities of the introduction and maintenance of anesthesia have already been described previously. The most important requirement for intraoperative management and maintenance of anesthesia is to avoid anything that could increase right-ventricular afterload or decrease contractility of the right ventricle, as both factors will ultimately lead to ischemia and right-sided heart failure (Figure 5). Perfusion of the right coronary artery is usually dependent on a pressure gradient between the aorta and the right ventricle, which, in the setting of increased RV afterload and decreased coronary blood flow, may lead to RV ischemia [59].

As one of the strongest inducers of pulmonary vasoconstriction is hypoxia, inspiratory oxygen administration should be set to a sufficiently high degree (FiO_2_ 0.6–1.0) to minimize the risk of hypoxic phases. This treatment can be supported by carefully performed recruitment maneuvers to largely eliminate inadequate ventilation-perfusion ratios [30]. Studies are yet to demonstrate if an intraoperative low-tidal-volume ventilation offers any benefits over “conventional” pressure-controlled ventilation, but the authors would recommend avoiding alveolar over-inflation in patients with pulmonary hypertension in order to set peak pressure as low as possible (6–8 mL/kg ideal body weight). In addition to hypoxia, acidosis and hypercapnia may also aggravate existing hypertension. Therefore, moderate hyperventilation (target PaCO_2_ of 30–35 mmHg) should be carried out under continuous blood gas analysis, but without allowing the pH value to fall below 7.4 [30, 41, 50]. Intraoperative management should ensure that depth of anesthesia and analgesia is always sufficient, as stress and pain during awareness may contribute to pulmonary vasoconstriction. Furthermore, intraoperative “standard measures” also include adequate thermal management. Hypothermia and shivering can considerably increase pulmonary pressure and should therefore be strictly avoided (Table 10).

Intraoperative fluid therapy should also be carried out rather restrictively and in a targeted manner, with adequate hemodynamic monitoring to optimize right-ventricular preload. It is very difficult to indicate general target values for these therapy forms that take account of the individual needs of this particular patient population. The target values for right-sided heart failure—for example, after heart transplantation—certainly cannot be applied to patients with chronic pulmonary hypertension [60, 61]. One possibility, which is favored by the authors, would be to consider the initial values measured during preoperative evaluation (right-heart catheter!) as target values and to initiate specific treatment in the event of deviations ±15–20%. Blaise et al. also recommend carrying out intraoperative management in a way that allows mean pulmonary artery pressure to fluctuate in a range of 15% above or below the initial value [50]. However, clinical trials have not yet collected sufficient data to substantiate this “target corridor.”

In general, it should be considered that patients with PH have low arterial pressure as a result of their disease and the specific therapy, and that the possibilities for compensation are very limited due to right-sided heart failure. *An essential goal is to maintain systemic blood pressure above pulmonary arterial pressures, thereby preserving coronary blood flow [59]. In their systematic review concerning the management of right-ventricular dysfunction, Price and coworkers found only low-quality evidence for the use of sympathomimetic (epinephrine, norepinephrine) and non-sympathomimetic vasopressors (vasopressin): They gave weak recommendations for norepinephrine in acute right-ventricular failure and for arginine-vasopressin in patients with vasodilatory shock and pulmonary vascular dysfunction, weak recommendations for low-dose dobutamine and levosimendan, and a strong recommendation for PDE-III-inhibitors [59].*

If an increase in pulmonary artery pressure occurs in the intra- or postoperative period and cannot be controlled by the symptomatic measures described above, specific medication should be induced immediately to reduce right-ventricular afterload and thus also the risk of right-sided heart failure. The required vasodilators can be administered both intravenously and by inhalation.

### 5.13. Intravenous Vasodilator Therapy

The administration of nitroglycerin, sodium nitroprusside, milrinone, dobutamine, or prostacyclin is recommended for intravenous vasodilation (for dosage see Table 11) [26, 30, 50, 62]. As the effect of these drugs is not limited to the pulmonary circulation and therefore also induces systemic vasodilation, their administration often causes a considerable decrease in systemic mean arterial pressure and involves the risk of right-ventricular perfusion pressure falling below a critical limit [62, 63]. In the case of hypotonic blood circulation, intravenous vasodilators should therefore only be administered with the greatest care. As a consequence of the complex co-effects of pulmonary and systemic resistance ratios, right-heart function, and patient-specific factors, the dosages listed in Table 11 can only serve as rough guidelines for clinical application. An individual concept is required for each patient.

The phosphodiesterase*3 *(PDE3) inhibitor milrinone has successfully been used in patients with pulmonary hypertension and cardiac surgery, and growing experience has been gathered to date in its administration [27, 62, 64]. It appears that the influence of milrinone on pulmonary resistance is more pronounced than the reduction of systemic resistance, which, together with a positive impact on myocardial contractility, makes it suitable for administration even in unstable circulatory situations [65]. Bolus administration with subsequent continuous infusion is recommended, although experience shows that the initial bolus administration should be omitted for patients with low mean pressure. Dobutamine also combines a positive inotropic effect with a reduction in pulmonary vascular resistance. However, the resulting tachycardia—which occurs at high dosages in particular—often limits therapy and, as a result, dobutamine administration is only recommended for mild to moderate elevations in pulmonary pressure [66]. The intravenous administration of sodium nitroprusside or prostacyclin can only be used for patients with a sufficiently high mean arterial pressure value. 

In 2010, sildenafil was approved for intravenous therapy of pulmonary arterial hypertension (PAH) and may be, for example, an attractive option for the perioperative management of patients who are treated with oral sildenafil.

### 5.14. Vasodilator Therapy by Inhalation

Selective pulmonary vasodilator therapy by inhalation offers several advantages over intravenous vasodilation. As alveoli and pulmonary capillaries are located in close proximity, the effect on the pulmonary vascular bed is limited, which avoids systemic vasodilation. Vasodilation therapy by inhalation can therefore also be administered to patients who already show limited right-ventricular function and for whom a further decrease of myocardial perfusion must be avoided. In addition, substances that are administered by inhalation only have an effect on ventilated lung areas, and the consecutive vasodilation in the ventilated areas *therefore may lead to a decrease* of the pulmonary shunt and improved oxygenation [67].

Several substances are currently available for vasodilator therapy by inhalation in patients with pulmonary hypertension (Table 11). Nitric oxide (NO) was the first substance to be used in inhalation therapy for diseases accompanied by a pathological elevation of pulmonary pressures [68]. Following diffusion into the smooth muscle cells, vascular relaxation is induced through the formation of cGMP. When it enters the bloodstream, it binds to erythrocyte hemoglobin within *milliseconds* [69]. However, in spite of its fast metabolization, the use of NO over an extended period of time, in particular in higher concentrations, is associated with a number of possible side effects and risks, for example, high percentage of nonresponder, rebound phenomena, and direct toxic effects to lung cells [70]. In a recently published paper Ibrahim et al. described increased blood concentrations of nitrite, nitrate, and s-nitrosyl hemoglobin in infants with pulmonary hypertension treated with inhaled NO. The authors speculate that these compounds may be carriers of NO-bioactivity and account for peripheral effects of inhaled NO [71]. In most cases, the use of NO must be accompanied by machine ventilation to ensure precise dosage. Although it can be administered using a tight-fitting breathing mask, this is difficult to apply in clinical practice. Patients only tolerate the maneuver for a very short period of time; the dosage is difficult to control and high personnel capacity is required [63].

Similar points can be made in relation to the equally short-acting prostacyclin which triggers vasodilation by elevating cAMP in the vascular muscle cells. Its half-life is only 2-3 minutes, and so the inhalation of prostacyclin also demands controlled ventilation [63]. The effectiveness of prostacyclin appears to be comparable with that of NO, although this has only been investigated in patients with lung failure [72]. In the perioperative setting, there exist several reports about the use of inhaled prostacyclin in patients with pulmonary hypertension. Schroeder reported about 5 patients with intraoperative impending right-ventricular failure, in all patients the prostacyclin inhalation was accompanied by a decrease of PVR and increase of cardiac output [73]. Haché reported about 35 patients with pulmonary hypertension, treated successfully with inhaled prostacyclin in the ICU or operation theatre [74]. 

**Figure 6:**
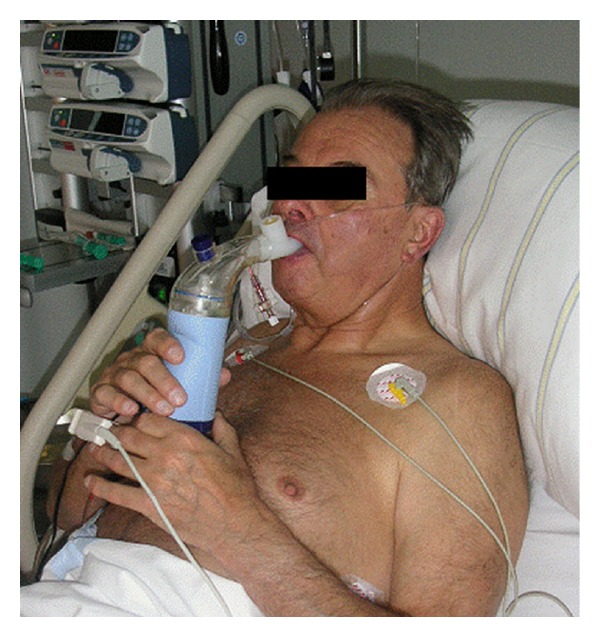
Preoperative inhalation of iloprost in spontaneous ventilation.

Due to its longer half-life of 20–30 minutes, the stable prostacyclin analog iloprost can be administered intermittently to ventilated and spontaneously breathing patients and therefore offers significant advantages over short-acting inhaled vasodilators (Figures 4(b) and 6) [63]. It is also approved for the treatment of pulmonary arterial hypertension. In addition, many articles have been published on off-label use in patients following cardiac surgical interventions, as well as those with chronic thromboembolic pulmonary hypertension and severe right-sided heart failure [75–77]. This substance should be administered using an ultrasonic nebulizer to ensure that particles of a specific size are inhaled and actually reach the alveoli. Gessler et al. have successfully demonstrated that inhaling iloprost with an ultrasonic nebulizer is much more efficient than using a jet nebulizer [78]. In relation to the inhalation of iloprost, it should be noted that, due to its longer half-life, systemic effects cannot be completely excluded when administering higher doses.The intraoperative use of inhaled iloprost is common and well documented in patients with pulmonary hypertension from different origins and cardiac surgery [75, 79–81]. Experiences with inhaled iloprost in patients undergoing noncardiac surgery are rare. Elliot et al. reported an inhalative treatment with iloprost in three women with pregnancy and caesarean section [82].

Another option for vasodilator therapy by inhalation, which, to date, has not been thoroughly described, is the inhalation of milrinone. In 2001, the working group headed by Haraldsson first described the inhaled administration of milrinone in combination with prostacyclin for cardiac surgical patients [83]. Dosage indications vary between 2 mg when testing pulmonary vascular responsiveness in cardiac transplantation candidates and 5 mg for application after cardiac surgical procedures [84, 85]. The data that is currently available is certainly not sufficient to recommend administration of this substance to patients with elevated pulmonary artery pressure. Although no side effects have been described in the articles published to date, the strong acid solution could potentially cause airway irritations and should therefore be diluted before administration [85]. Even so, combining a phosphodiesterase inhibitor with iloprost may still be worthy of consideration and may prove effectiveness in patients who show little or no response to the administration of iloprost alone, or who have an acute and dramatic elevation of pulmonary artery pressures [86]. In cardiac surgery, there exist various reports about the use of inhaled milrinone, which confirm the first impression, that inhalation of the PDE3-inhibitor induces pulmonary vasodilation without significant side effects [87]. First experiences with the simultaneous inhalation of prostacyclin and milrinone were published in 2011 by Huang and coworkers [88].

A new option as inhalative therapy of pulmonary hypertension is inhaled treprostinil, a chemically stable tricyclic benzindene prostanoid which was initially studied as a continuous subcutaneous infusion [89, 90]. Due to the longer half-life of treprostinil as compared to iloprost, there was a strong rationale for developing treprostinil for inhalation [91]. The results of the first pilot studies with inhaled treprostinil compared to inhaled iloprost were published by Voswinckel and coworkers. It was found that inhalation of both drugs resulted in a comparable reduction of PVR, but the peak effect of inhaled treprostinil occurred later than after inhaled iloprost and the duration of treprostinil effect [91, 92]. Since 2009, inhaled treprostinil is approved by FDA (TM: Tyvaso). Currently there are no published reports about the use of inhaled treprostinil in the perioperative setting; the pharmacologic profile of this drug makes it attractive for the perioperative use in patients with pulmonary hypertension for a limited period of time in the future.

It has been shown by the study of Loh and colleagues that inhalation of nitric oxide in patients with reactive pulmonary hypertension secondary to left-ventricular failure, inhalation of NO, causes reciprocal changes in the PVR (decrease) and left-ventricular filling pressure (increase) [93]. This results can be transferred to all inhaled vasodilators; they should not be administered to patients with decompensated left-sided heart failure because, in the event of massive pulmonary back pressure caused by left-ventricular afterload reduction, the selective pulmonary vasodilation may trigger an acute pulmonary edema [94].

### 5.15. Postoperative Recovery and Analgesic Therapy

Patients with pulmonary hypertension are at risk of developing elevated pulmonary pressure and right-sided heart failure not only during the perioperative phase itself, but also in the postoperative course. These patients should therefore be placed under intense postoperative monitoring for a period appropriate to the degree of surgical trauma; the target monitoring time should be between 24 hours for small interventions and several days for major procedures (abdominal and thoracic surgery, major urological interventions). Depending on the patient's initial condition (functional classification), hemodynamic monitoring may need to be continued postoperatively until pulmonary pressures, and right-sided heart functions have stabilized at the preoperative level [27].

In this phase, sufficient analgesic therapy can make a decisive contribution to the avoidance of elevated pulmonary pressures. In the ideal case, analgesic therapy in the form of continuous regional anesthesia can be designed in a way that avoids higher doses of opioid-based analgesics. The basic treatment of patients with pulmonary hypertension therefore includes daily visits by pain management nurses. 

The specific therapy for pulmonary hypertension should be resumed at the preoperative dosage as soon as possible. In the postoperative course, it is also advisable to treat pressure elevations with iloprost inhalation, which can also be administered intermittently due to its long half-life.

## 6. Conclusions for Clinical Practice

Pulmonary hypertension is a major reason for elevated perioperative morbidity and mortality, even in noncardiac surgical procedures. Patients should be thoroughly prepared for the intervention and allowed plenty of time for consideration. All specialty units involved in treatment should play a role in these preparations. After selecting each of the suitable individual anesthetic and surgical procedures, intraoperative management should focus on avoiding all circumstances that could contribute to exacerbating pulmonary hypertension (hypoxemia, hypercapnia, acidosis, hypothermia, and hypervolemia). A method of treating elevations in pulmonary pressure with selective pulmonary vasodilation by inhalation should be available intraoperatively, in addition to invasive hemodynamic monitoring. During the postoperative phase, patients must be monitored continuously and receive sufficient analgesic therapy over an adequate period of time. All in all, perioperative management of patients with pulmonary hypertension presents an interdisciplinary challenge that requires the adequate involvement of anesthetists, surgeons, pulmonologists, and cardiologists alike.

## Figures and Tables

**Table 1:** Classification of pulmonary hypertension (Dana-point [7]).

(1) Pulmonary artery hypertension	
(1.1) Idiopathic (IPAH)	
(1.2) Hereditary (HPAH)—BMPR2, ALK-1, endoglin	
(1.3) Drug and toxin induced	
(1.4) Associated pulmonary artery hypertension (APAH)	
(1.4.1) Connective tissue disorders	
(1.4.2) HIV infection	
(1.4.3) Portal hypertension	
(1.4.4) Congenital heart diseases	
(1.4.5) Schistosomiasis	
(1.4.6) Chronic hemolytic anemia	
(1.4.7) Persistent newborn pulmonary hypertension	
(1.5) Pulmonary veno-occlusive disease and/or pulmonary capillary hemangiomatosis (PCH)	
(2) Pulmonary hypertension caused by left-heart disease	
(2.1) Systolic dysfunction	
(2.2) Diastolic dysfunction	
(2.3) Valve disease	
(3) Pulmonary hypertension secondary to pulmonary diseases and/or hypoxemia	
(3.1) Chronic obstructive pulmonary disease	
(3.2) Interstitial pulmonary disease	
(3.3) Other pulmonary diseases with mixed restrictive and obstructive patterns	
(3.4) Sleep-disordered breathing	
(3.5) Alveolar hypoventilation disorders	
(3.6) Chronic high-altitude exposure	
(3.7) Developmental abnormalities	
(4) Chronic thromboembolic pulmonary hypertension (CTEPH)	
(5) Pulmonary hypertension with unclear or multifactorial mechanisms	
(5.1) Hematological disorders: myeloproliferative and splenectomy	
(5.2) Systemic disorders: sarcoidosis, pulmonary Langerhans cell histiocytosis, lymphangioleiomyomatosis, neurofibromatosis, and vasculitis	
(5.3) Metabolic disorders: glycogen storage disease, Gaucher's disease, and thyroid disorders	
(5.4) Others: tumoral obstruction, fibrous mediastinitis, and chronic renal failure with dialysis	

**Table 2:** Hemodynamic characteristics in patients with pulmonary hypertension (mod. [8]).

Definition	Characteristics	Etiology
*Pulmonary*	PAPm ≥ 25 mmHg	all
*Hypertension (PH)*	CO normal or reduced	
	PAPm ≥ 25 mmHg PCWP ≤ 15 mmHg CO normal or reduced	*Group 1*: Pulmonary artery hypertension (IPAH)
*Precapillary PH*		*Group 3*: PH with pulmonary diseases and/or hypoxemia
		*Group 4*: CTEPH
		*Group 5*: PH with unclear or multifactorial mechanisms
	PAPm ≥ 25 mmHg	
*Postcapillary PH*	PCWP > 15 mmHg	*Group 2*: PH with left-heart diseases
	CO normal or reduced	
(a) passive	TPG ≤ 12 mmHg	
(b) reactive	TPG > 12 mmHg	

PAPm: mean pulmonary arterial pressure; CO: cardiac output; PCWP: pulmonary capillary wedge pressure; TPG: transpulmonary gradient (= PAPm − PCWP).

**Table 3:** Clinical findings in patients with pulmonary hypertension (mod. [8]).

(i) Dyspnea (during stress/at rest)/cyanosis
(ii) Fatigue
(iii) Dizziness
(iv) Synkope
(v) Thoracic pain
(vi) Palpitations
(vii) Orthopnea
(viii) Cough
(ix) Croakiness
(x) Abdominal tension
(xi) Peripheral Edema/Ascites
(xii) Hepatomegaly

**Table 4:** Therapy of pulmonary hypertension: approved drugs (mod. [8]).

Drug		Dosage	Side effect
Bosentan	Endothelin receptor antagonist	2 × 62,5–125 mg/d po	Increase of liver enzymes edema
Ambrisentan	Selective endothelin-A Receptor-antagonist	1 × 5–10 mg/d po	Increase of liver enzymes edema
Sildenafil	PDE-5 inhibitor	3 × 20 mg/d po	Reflux esophagitis
Tadalafil	PDE-5 inhibitor	1 × 40 mg/d po	Headache and pain in the limbs
Iloprost	Prostacyclin-analog	6–9 × 2,5/5 *μ*g inhalative	Hypotension and flush
Treprostinil	Prostacyclin-analog	1,25–22,5 ng/kg/min s.c.	Local pain

**Table 5:** Functional classification of pulmonary hypertension (WHO 1998) [28].

Class I	Patients with pulmonary hypertension but without resulting limitation of physical activity. Ordinary physical activity does not cause dyspnea or fatigue, chest pain or near syncope.
Class II	Patients with pulmonary hypertension resulting in slight limitation of physical activity. They are comfortable at rest. Ordinary physical activity causes undue dyspnea or fatigue, chest pain or near syncope.
Class III	Patients with pulmonary hypertension resulting in marked limitation of physical activity. They are comfortable at rest. Less than ordinary physical activity causes undue dyspnea or fatigue, chest pain or near syncope.
Class IV	Patients with pulmonary hypertension with inability to carry out any physical activity without symptoms. These patients manifest signs of right-heart failure. Dyspnea and/or fatigue may even be present at rest.

**Table 6:** ECG-findings in chronic right-heart failure.

(i) Sinus tachycardia	
(ii) Vertical/right type	
(iii) Positive Sokolow-Lyon index for right-ventricular hypertrophy	
(iv) p-pulmonalis	
(v) S_I_/S_II_/S_III_-Type and S_I_/Q_III_-Type	
(vi) Incomplete or complete right bundle branch block	
(vii) Right-ventricular repolarization disorder	

**Table 7:** Right-heart catheterization: parameters for evaluation.

(i) Pulmonary arterial pressure (PAP; systolic, diastolic, and mean)
(ii) Pulmonary capillary wedge pressure (PCWP)
(iii) Right-ventricular pressure (RVP)
(iv) Right-atrial pressure (RAP)
(v) Cardiac output (CO); cardiac index (CI)
(vi) Oxygen saturation (systemic arterial; pulmonary arterial)
(vii) Pulmonary vascular resistance (PVR)

**Table 8:** Intraoperative monitoring: recommendation for patients with PH.

(i) Basic monitoring
(a) ECG
(b) SaO_2_
(c) End-expiratory CO_2_
(d) Invasive blood pressure
(e) Optional: stroke volume variation (SVV)
(ii) Extended monitoring
(a) Pulmonary arterial catheter
(b) Transesophageal echocardiography (TEE)
(c) ScvO_2_

**Table 9:** Authors recommendations for the human and structural and technical requirements for the perioperative management of patients with severe pulmonary hypertension.

(i) Established cooperation with cardiologists and pulmonologists
(ii) Access to specific medication for the treatment of pulmonary hypertension
(iii) Experiences in all procedures of general and regional anesthesia
(iv) Experiences in dealing with pulmonary arterial catheterization and the use of inhaled drugs for selective pulmonary vasodilation
(v) Intraoperative transesophageal echocardiography
(vi) Hemodynamic monitoring in critical care
(vii) Specific educational program for “pulmonary hypertension”
(viii) Consultants with special experiences in the treatment of pulmonary hypertension
(ix) Regular pain visits and/or pain nurses for the perioperative pain therapy

**Table 10:** Intraoperative “basic treatment” to avoid an increase of pulmonary arterial pressure (mod. [30, 31, 39, 48, 59]).

(i) “luxury” oxygenation with inspiratory FiO_2_ 0,6–1,0	
(ii) Moderate hyperventilation (goal: PaCO_2_ 30–35 mmHg)	
(iii) Avoidance of metabolic acidosis (pH > 7,4)	
(iv) Recruitment maneuver to avoid ventilation/perfusion mismatch	
(v) Low-tidal-volume ventilation to avoid overinflation of aveoli (goal: 6–8 mL/kg ideal body weight)	
(vii) Temperature management to maintain body temperature of 36-37°C	
(viii) “goal-directed” fluid and volume therapy with hemodynamic monitoring	

**Table 11:** Specific interventions for therapy of intra- and/or postoperative increase of pulmonary arterial pressure (mod. [30, 31, 38, 39, 48, 59, 60]).

Reduction of right-ventricular afterload:	
Intravenous vasodilation	
(1) Milrinone	50 *μ*g/kgBW bolus, followed by 0,5–0,75 *μ*g/kgBW/min continuously
(2) Dobutamine	2–5 *μ*g/kgBW/min continuously
(3) Prostacyclin	4–10 ng/kgBW/min continuously
(4) Na-nitruprusside	0,2–0,3 *μ*g/kgBW/min continuously
(5) Nitroglycerine	2–10 *μ*g/kgBW/min continuously
Pulmonary-selective inhalative vasodilatation	
(1) Iloprost	5–10 *μ*g for 10–15 min (by untrasonic nebulizer)
(2) Nitrogen monoxide	0,5–20 ppm continuously
(3) Prostacyclin	30–40 ng/kgBW/min continuously
(4) Milrinone	2 mg (−5 mg) for 10–15 min (diluted in 10–15 mL NaCl0,9%)
